# Chronic Maternal Low-Protein Diet in Mice Affects Anxiety, Night-Time Energy Expenditure and Sleep Patterns, but Not Circadian Rhythm in Male Offspring

**DOI:** 10.1371/journal.pone.0170127

**Published:** 2017-01-18

**Authors:** Randy F. Crossland, Alfred Balasa, Rajesh Ramakrishnan, Sangeetha K. Mahadevan, Marta L. Fiorotto, Ignatia B. Van den Veyver

**Affiliations:** 1 Department of Obstetrics & Gynecology, Baylor College of Medicine, Houston, TX, United States of America; 2 Jan and Dan Duncan Neurological Research Institute at Texas Children’s Hospital, Houston, TX, United States of America; 3 Interdepartmental Graduate Program in Translational Biology and Molecular Medicine, Baylor College of Medicine, Houston, TX, United States of America; 4 USDA/Agricultural Research Service Children’s Nutrition Research Center, Houston, TX, United States of America; 5 Department of Molecular and Human Genetics, Baylor College of Medicine, Houston, TX, United States of America; Universidade do Estado do Rio de Janeiro, BRAZIL

## Abstract

Offspring of murine dams chronically fed a protein-restricted diet have an increased risk for metabolic and neurobehavioral disorders. Previously we showed that adult offspring, developmentally exposed to a chronic maternal low-protein (MLP) diet, had lower body and hind-leg muscle weights and decreased liver enzyme serum levels. We conducted energy expenditure, neurobehavioral and circadian rhythm assays in male offspring to examine mechanisms for the body-weight phenotype and assess neurodevelopmental implications of MLP exposure. C57BL/6J dams were fed a protein restricted (8%protein, MLP) or a control protein (20% protein, C) diet from four weeks before mating until weaning of offspring. Male offspring were weaned to standard rodent diet (20% protein) and single-housed until 8–12 weeks of age. We examined body composition, food intake, energy expenditure, spontaneous rearing activity and sleep patterns and performed behavioral assays for anxiety (open field activity, elevated plus maze [EPM], light/dark exploration), depression (tail suspension and forced swim test), sociability (three-chamber), repetitive (marble burying), learning and memory (fear conditioning), and circadian behavior (wheel-running activity during light-dark and constant dark cycles). We also measured circadian gene expression in hypothalamus and liver at different Zeitgeber times (ZT). Male offspring from separate MLP exposed dams had significantly greater body fat (P = 0.03), less energy expenditure (P = 0.004), less rearing activity (P = 0.04) and a greater number of night-time rest/sleep bouts (P = 0.03) compared to control. MLP offspring displayed greater anxiety-like behavior in the EPM (P<0.01) but had no learning and memory deficit in fear-conditioning assay (P = 0.02). There was an effect of time on *Per1*, *Per 2* and *Clock* circadian gene expression in the hypothalamus but not on circadian behavior. Thus, transplacental and early developmental exposure of dams to chronic MLP reduces food intake and energy expenditure, increases anxiety like behavior and disturbs sleep patterns but not circadian rhythm in adult male offspring.

## Introduction

Maternal diet has a significant impact on fetal growth, with maternal malnutrition being a major cause of intrauterine growth restriction (IUGR) in developing countries [1]. IUGR or low birthweight increases the risk for cardiovascular and metabolic diseases in adulthood [2–4]. The WHO estimates that around 300 million people worldwide will suffer from metabolic disorders by 2025 [5]. Exposure to a maternal low protein diet (MLP) during gestation and/or lactation has been used extensively as a model of fetal malnutrition, with MLP offspring found to be at increased risk of cardiovascular and metabolic disorders in adulthood [2, 3]. Recently, it has been shown in mice that maternal dietary restriction has metabolic and neurobehavioral effects on offspring [6, 7]. Similar results have been found in rat and human studies [8–10]. However, few studies have addressed the effects of chronic protein malnutrition starting well before gestation.

Neural development begins in the early stages of gestation, a critical developmental period demonstrated to be sensitive to environmental, physiological and nutritional modifications [11–13]. Offspring exposed to MLP only during gestation and weaning exhibit signs of anxiety, depression, and impaired learning and memory [14, 15]. In this context, limbic system components such as the hypothalamus, hippocampus, and amygdala, are of particular interest given their potential involvement in MLP-associated neurobehavioral and physiological pathologies [16–19]. MLP offspring were found to have impaired hippocampal and hypothalamic neuronal proliferation [20], suggesting a direct effect of gestational MLP on the development and function of these regions, but effects of chronic MLP diet on these systems are unknown.

Furthermore, the hypothalamus plays a central role in the establishment and maintenance of circadian rhythms [21]. Also, all circadian clocks, including the peripheral clocks, are regulated by a master clock in the suprachiasmatic nucleus (SCN) of the hypothalamus [22]. Altered circadian rhythms have been demonstrated to precipitate cardiovascular, metabolic and mood disorders [23, 24]. Circadian gene expression changes in liver have been previously reported in various species exposed to a high-fat diet [25, 26]. However, little is known about the effects of MLP on circadian rhythms in exposed offspring. Differences in behavioral and circadian patterns can significantly impact energy metabolism and activity.

We previously showed that male offspring from dams chronically fed MLP from 4 weeks prior to pregnancy onwards display reduced body weight, reduced size of specific hind limb muscles, lower serum levels of liver enzymes from weaning up to one year of age and altered expression of cohesin-mediator complex genes which may play a role in epigenetic regulation [27].

In the current study, we assessed whole body composition and energy expenditure, together with an extensive neurobehavioral examination of male offspring born to dams chronically fed a low protein diet. The goal was to investigate how chronic low protein diet in dams influences metabolism, circadian rhythm and neurobehavior in male mice offspring. We found that offspring of dams on low protein diet were smaller, had higher body fat content, showed nocturnal hypoactivity and lower levels of energy expenditure (EE). These offspring had no circadian rhythm alterations but exhibited mild anxiety related behavioral differences.

## Materials and Methods

### Animals and Experimental Design

C57BL6/J mice were obtained from Center of Comparative Medicine at Baylor College of Medicine (BCM) and colonies were maintained at BCM. Dams were fed a protein-restricted diet before and during gestation, and throughout lactation as previously described [27]. Briefly, female C57BL6/J mice at 3–4 weeks of age were divided into two groups, MLP (8% protein; TD93033, Harlan Teklad) and control (20% protein; TD91352, Harlan Teklad) and were fed their respective diets *ad libitum* from four weeks prior to mating with C57BL6/J males until pups were weaned. The diets were isocaloric, with carbohydrate replacing protein in the low protein diet. As carbohydrate and protein have a similar energy density, the low protein diet does not have to be a high fat diet to be isocaloric.

Upon detecting a copulatory plug, pregnant females were housed individually. Litters were culled to a maximum of 6 male pups/dam on postnatal day (PND) 3. On PND21, male pups were weaned to a laboratory non-purified diet (20% protein; PicoLab Rodent Diet 20–5053, Lab diet) and housed individually. These singly housed pups were considered separate units for purpose of data analysis. Offspring from 2–5 litters were used in all the tests. Nestlets were provided to all offspring for environmental enrichment purposes. The study protocol was approved by the Institutional Animal Care and Use Committee (IACUC) at BCM. All experiments were conducted according to institutional and governmental regulations concerning the ethical use of animals in research. All animal facilities are approved by the Association for Assessment and Accreditation for Laboratory Animal Care International (AAALAC).

### Energy balance

Measurements of body weight composition, energy balance, food intake and activity were performed in the Mouse Metabolic Research Unit at the USDA/ARS Children’s Nutrition Research Center BCM at 12 weeks of age on 7 male offspring of each group.

#### Food consumption, energy expenditure and activity analysis

The Comprehensive Laboratory Animal Monitoring System (CLAMS) (Columbus Instruments) was used to monitor food intake, energy expenditure (EE) and activity in MLP and control mice (n = 7 each) as described previously [28]. The mice were first acclimated to the CLAMS cages for three days followed by three experimental days when food intake, EE, and activity were monitored concomitantly. Food and water were available *ad libitum* throughout. Room temperature was maintained at 23.5°C with a 12h light/dark cycle. Data was analyzed using the CLAMS data eXamination Tool (CLAX) (version 2.1.0; Columbus Instruments). The amount of time the mice spent completely inactive, or “sleeping”, was determined from the high-frequency recording of cage activity as described by Pack *et al*. [29]; 1 epoch was defined as a period of 60 seconds in which no movement was detected. Because the CLAMS cannot distinguish rest stage from active REM sleep, we refer to these parameters as “resting/sleeping” in this study.

#### Body composition

The fat and lean content of the mice were measured with a Quantitative Magnetic Resonance (QMR) analyzer (EchoMedical, Houston Texas) at the completion of the CLAMS measurements.

### Neurobehavioral Analysis

All neurobehavioral assays were carried out in the Neurobehavioral core facility of the BCM Intellectual and Developmental Disabilities Research Center (IDDRC). The mice were acclimated to the procedure room 30 minutes before commencing the behavioral test. Unless otherwise noted, the lighting in the procedural room was 800–900 lux intensity, and the background noise maintained at 60±5 dB with the use of a white-noise generator. All the assays were conducted at approximately the same time of the day in batches, but for each batch, approximately equal numbers of controls and MLP-exposed offspring were simultaneously analyzed. Behavioral assays were simultaneously conducted on groups of 20 male MLP-exposed offspring and 20 control offspring at 8–12 weeks of age, except where other numbers are specified.

#### Elevated plus maze (EPM)

Mice were evaluated for anxiety-like behavior on a plus-shaped platform elevated 50 cm above the floor as previously described [30]. The ANY-maze software was used to track the mouse position in the elevated plus maze for 10 minutes. The number of visits and the time spent in the open arms were recorded as measures of anxiety.

#### Tail suspension test (TST)

A total of 31 MLP and 13 control mice were evaluated by tail suspension test [31] to study depressive behavior. Movement of the mice was monitored for six minutes in real-time using charge coupled device (CCD) cameras linked with ANY-Maze software. The data was compiled in two-minute bins, and assessed for the immobile time.

#### Forced swim Test (FST)

Mice were evaluated for depression-like behavior in the forced swim test [32]. Mice were placed into the water chamber for six minutes, and movements were monitored in real-time with CCD cameras interfaced with the ANY-Maze software. The data was compiled in two-minute bins and assessed for immobile time.

#### Three-chamber test (3CH)

Social behavior was evaluated using the three-chamber test [33]. The mice were acclimated to the unit and placed in the middle chamber. Social preference was measured by providing the test mouse a choice of exploring a chamber containing a novel mouse or containing a novel object. The novel mouse was acclimated to the cups in the apparatus for one hour per day for two days prior to testing and was used only once per day in the test. The location for the novel mouse was alternated between the left and right sides of the social test box between subjects. The amount of time spent by the test mouse sniffing each cup was scored with the ANY-maze software program.

#### Marble burying test

Repetitive behavior was assayed using the marble burying test [34]. Twenty clean marbles were evenly distributed on the surface of 5-cm deep corncob bedding in a standard housing cage. The mice were acclimated to the test room for 30 minutes and then placed in the cage containing the marbles. They were allowed to explore for 30 minutes and the number of buried marbles were enumerated.

#### Fear conditioning assay

Fear-associated learning and memory was evaluated with a standard contextual and cued fear-conditioning assay as previously described [30, 35]. Each animal was continually monitored with a digital CCD camera (ACT-VP-02). FreezeFrame 3 software was used to quantitate the percentage of freezing time, as well as to implement automated protocols for the fear conditioning assay.

#### Open-field activity (OFA)

Mice were assessed for exploratory and anxiety-like behavior using in open-field assay according to a standard protocol [36]. The following parameters were assessed: total distance traveled, distance traveled in the center, time spent being mobile, speed of motion, stereotypy, vertical activity, and revolutions. Data was collected and analyzed in 10-minute bins.

#### Light/dark exploration

The light/dark exploratory assay was conducted per standard protocol [37] to measure anxiety-related behavior using a commercially available apparatus (Accuscan Instruments, Inc.) containing an acrylic chamber divided into a light and dark compartment, specially designed to fit into a VersaMax monitor base (Cat# 41808; Accuscan Instruments, Inc.). Data was collected with VersaMax Software (version 4.12-1AFE) and analyzed in one minute bins to quantify total number of transitions between compartments, time spent in the light compartment and latency to entry in the dark compartment.

### Circadian Rhythm Assays

#### Wheel running assay

The wheel-running assay was used to evaluate circadian behavior [38] using the Mini-Mitter system (Respironics Company, Inc.) with VitalView and ActiView software version 4.2. Mice (MLP, n = 15; Control, n = 9) were single-housed in specialized wheel-running cages with food and water provided *ad libitum*. Briefly, actograms were analyzed to determine periodicity (Tau) of active cycles during a 12-hour light phase followed by a 12-hour dark phase (L:D) in the initial 2 weeks of observation and during constant darkness (D:D) for another 2 weeks. The phase shift was determined by the difference in periodicity between the L:D and D:D phases. Mice are typically more active at night; hence we refer to these phases as light/rest and dark/active throughout the results and discussion.

#### Circadian gene expression studies

At 18–20 weeks of age, offspring housed under a L:D cycle for more than two weeks were randomly assigned to one of four tissue collection Zeitgeber time (ZT) points: ZT0, ZT6, ZT12, or ZT18. Animals were deeply anesthetized with isoflurane and rapidly decapitated. Hypothalamus was harvested and flash frozen in liquid N_2_, and stored at -80°C until needed. Tissues were homogenized with a pestle (P7339-901; Argos Technologies). Total RNA was isolated using miRNeasy Mini Kits (217004; Qiagen) following manufacturer’s instruction. An on-column DNase treatment was performed using RNase-Free DNase Set (79254; Qiagen). cDNA was synthesized using qScript cDNA Supermix (95048; Quanta Biosciences). qRT-PCR was performed using PerfeCTa^®^ SYBR^®^ Green FastMix (95072; Quanta Biosciences) on the Bio-Rad CFX Connect Real-Time instrument. The ΔΔ*C*_t_ method was used for analysis with *18s* r*RNA* and *Actin* as the housekeeping genes. The primer sequences are shown in S1 Table.

### Statistics

Statistical analysis was performed considering each offspring as a single unit, as they were housed as one mouse/cage. Energy expenditure and food intake data were analyzed by ANCOVA using lean and fat mass or body weight as covariates. Tukey’s test was applied to *post hoc* analyses. Two-tailed student t-test was used to analyze data from resting/sleeping analysis, Elevated Plus Maze, Forced Swim test, 3-Chamber test, Marble Burying test, Open Field Activity test and Light/Dark Exploration test. The data sets for Tail Suspension test, Fear Conditioning Assay and Circadian Wheel Running assay where sample sizes were different were first run for D’Agostino & Pearson omnibus normality and Shapiro-Wilk normality test followed by analysis as a non-Gaussian distribution using the Mann-Whitney U test. Tau values were analyzed by the F test for variances. Circadian gene expression data was analyzed by 2-way ANOVA with Bonferroni correction. GraphPad Prism version 6 was used for statistical analysis. For all analyses, a P-value of <0.05 was considered statistically significant. All data are presented as mean ± standard error of the mean.

## Results

### Offspring exposed to chronic MLP diet show increased body fat, reduced energy expenditure, and disrupted rest/sleep pattern

**Fig 1:**
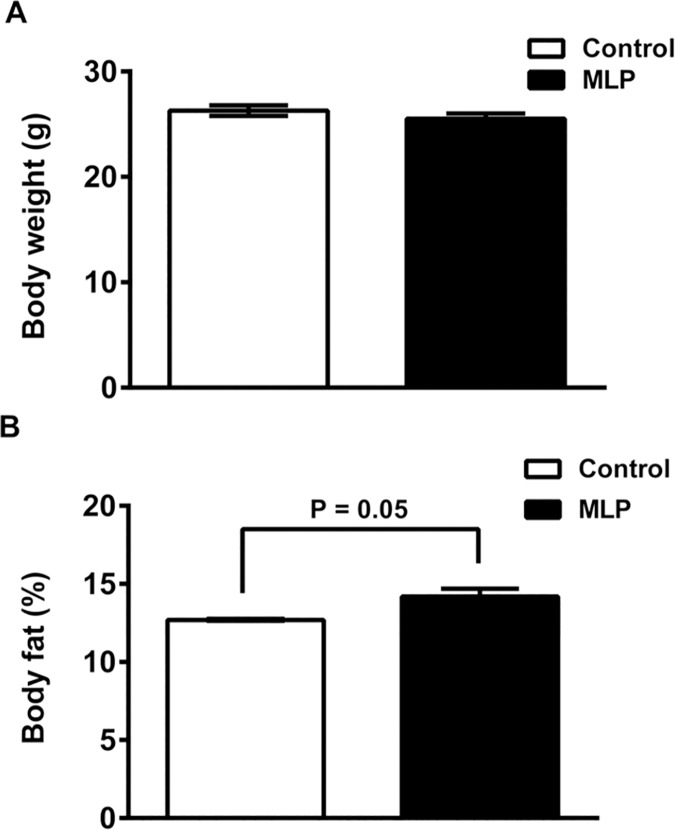
Body composition. (A): Body weight; (B) Body fat as percent of total body mass in male offspring (8–12 weeks age) from dams exposed to either control or MLP diet (n = 7 each). Bars are mean ± SEM and P<0.05 statistically significant by student t-test.

Because we have shown previously that adult male MLP offspring had lower overall body weight and decreased weight of selected hind leg muscles [27], we wished to determine whether this was also associated with alterations in overall body composition and its impact on energy homeostasis. Body weights were similar between MLP and control offspring at this single time point (12 weeks) (P = 0.06) (Fig 1A) which is in line with the similar weight gain over time found in our previous study [27]. However, a greater proportion of the body weight of MLP offspring was derived from fat (MLP, 14.4 ± 0.5% body weight; control, 12.7 ± 0.5% body weight; P = 0.05) (Fig 1B).

We next compared food intake and energy balance. Overall, total daily food intake, adjusted for variations in body weight was similar between control and MLP offspring (P = 0.28), with MLP displaying a lower intake during the dark phase (P<0.1; Table 1); with the omission of one outlier, the difference was significant (2.17 ± 0.11, P <0.001). However, total daily energy expenditure (EE) was significantly lower in MLP mice (P<0.02) primarily during the dark phase (P<0.01) (Table 1). There was no dampening in the amplitude of oscillation in energy uptake as there was no significant difference between mean values in the ratio of light to dark food intake for control (0.477 ± 0.040) and MLP offspring (0.528 ± 0.037). In MLP offspring, the dark phase was also associated with a small but significantly lower respiratory exchange ratio (RER) (P<0.001) (Table 1).

**Table 1:** Food intake and energy expenditure in 12-wk-old MLP and Control offspring showing values over 24 hours, and the distribution between light and dark phases.

		Control	MLP	P
n		7	7	
Food Intake^1^, g				
	24 h	4.07 ± 0.22	3.71 ± 0.22	0.28
	12 h light	1.27 ± 0.08	1.33 ± 0.08	0.59
	12 h dark	2.81 ± 0.16	2.38 ± 0.16	0.10
Total Energy expenditure (EE)^2^, kcal				
	24 h	9.93 ± 0.18	9.05 ± 0.18	<0.02
	12 h light	4.41 ± 0.10	4.10 ± 0.10	0.08
	12 h dark	5.53 ± 0.09	4.95 ± 0.09	<0.01
Respiratory Exchange Ratio (RER)				
	24 h	0.868 ± 0.004	0.861 ± 0.004	0.53
	12 h light	0.819 ± 0.008	0.830 ± 0.008	0.12
	12 h dark	0.917 ± 0.003	0.892± 0.003	<0.001

^1^Values are least square means, adjusted for body weight, ± SEM

^2^ Values are least square means, adjusted for lean and fat mass, ± SEMP<0.05 was considered statistically significant by ANCOVA with Tukey’s post hoc test.

In addition to feeding, spontaneous activity and sleeping are two behaviors that are known to differ in frequency between the dark and light phases, and which could have a significant influence on EE. The CLAMS system provides quantitative measures of total activity in the horizontal plane (X total) comprised of ambulatory activity (X ambulatory) and non-ambulatory activity associated with stereotypical behaviors such as grooming and twitching (X fidgeting), and rearing in the vertical plane (Z total). Although all mean values were numerically smaller in MLP mice, only Z total counts were significantly different (P = 0.04, Table 2) due exclusively to less rearing in the MLP mice during the dark phase. There were no differences between groups in the circadian pattern of any other measure of activity.

**Table 2:** Spontaneous cage activity, measured as infra-red beam breaks in 12-wk-old MLP and Control offspring; values over 24 hours, and the distribution between light and dark phases are shown.

	Control	MLP	P
n	7	7	
24 hours			
X total	41708 ± 226	37801 ± 241	0.26
X ambulatory	21097 ± 155	18542 ± 166	0.28
X fidgeting	20611 ± 821	19259 ± 878	0.28
Z total	6377 ± 656	4270 ± 701	0.05
Total activity (X total +Z total)	48085 ± 284	42070 ± 303	0.17
Light/resting phase			
X total	8493 ± 410	8053 ± 438	0.48
X ambulatory	3163 ± 265	3144 ± 265	0.96
X fidgeting	5329 ± 194	4910 ± 208	0.16
Z total	733 ± 147	477 ± 157	0.26
Total activity (X total + Z total)	9225 ± 563	8531 ± 563	0.38
Dark/Active phase			
X total	33215 ± 206	29747 ± 223	0.28
X ambulatory	17933 ± 145	15398 ± 155	0.25
X fidgeting	15282 ± 723	14349 ± 772	0.39
Z total	5645 ± 547	3792 ± 585	0.04
Total activity (X total + Z total)	38860 ± 257	33539 ± 275	0.18

Values are means ± SEM. P<0.05 was considered statistically significant by ANCOVA with Tukey’s post hoc test

**Fig 2:**
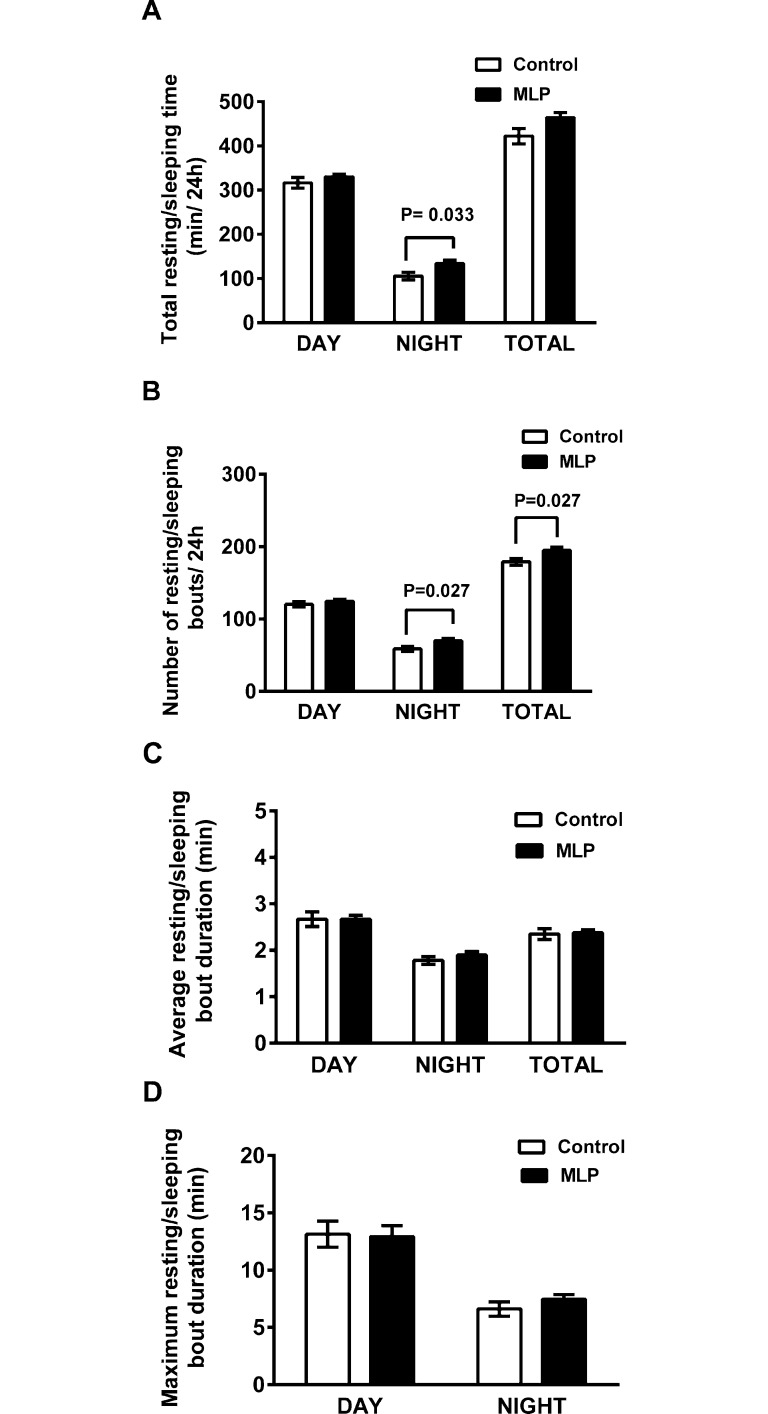
MLP male offspring mice display dysfunctional night time resting/sleeping patterns. (A): Total resting/sleeping time/day; (B): Sleep bouts/day; (C): Sleep bout duration; (D): Maximum sleep bout duration (n = 7/group, 12 weeks age). Data shown is mean ± SEM with P<0.05 considered statistically significant by student t-test.

Given the lower rearing activity during the dark phase, we assessed the amount of time mice spent resting/sleeping. Over 24 hours, MLP offspring showed a non-significant trend for overall more time resting/sleeping (P = 0.07). This trend results from MLP offspring spending significantly more time resting/sleeping during the dark phase (P = 0.033), when mice are normally more active, but there was no difference between the groups in the time spent resting/sleeping during the light phase (Fig 2A). The increased amount of time spent resting/sleeping in the MLP offspring was the result of a significantly greater number of rest/sleep bouts, primarily during the night (P = 0.027, Fig 2B) compared to control offspring with no difference in the mean duration (Fig 2C) or maximum length of rest/sleep bouts (Fig 2D).

Taken together, the data suggest that the altered behavior of MLP offspring during the dark phase described above contributed to their lower overall EE. This could be due to circadian activity abnormality or behavioral changes, including anxiety or depression, which has been reported before [39, 40]. Therefore, we explored circadian activity including gene expression of circadian genes, as well as neurobehavior in the MLP-exposed offspring.

### Disrupted rest/sleep pattern in MLP exposed offspring is not associated with circadian activity and gene expression

**Fig 3:**
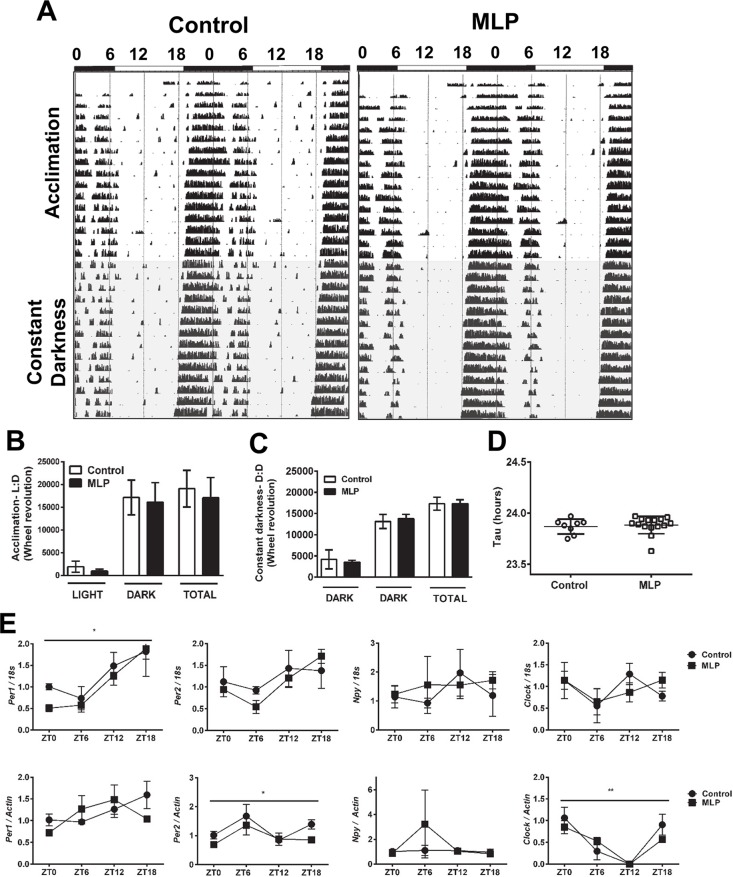
Circadian physical activity and gene expression is not affected in MLP male offspring mice. (A) Representative double-plotted actogram for control (left panel) and MLP (right panel) offspring. Zeitgeber times (ZT) (0, 6, 12, 18) are represented on top. Black and white blocks below ZT’s indicate dark and light conditions, respectively. Mice were individually housed first in 12 h light:12 h dark for acclimation (L:D) (white background, top half) then in constant darkness (D:D) (gray background, bottom half). Black notches represent activity. The total wheel revolutions during final 10 days of the (B) acclimation (Control, n = 9; MLP, n = 15), (C) constant darkness (Control, n = 8; MLP, n = 15), and (D) Periodicity (Tau) (Control, n = 8; MLP, n = 15) is shown. (E) Total RNA (mRNA) from hypothalamus of MLP and control offspring (n = 3/time point/group) after two weeks housing under L:D cycle were extracted at ZT 0, 6, 12, or 18. Data shown is relative gene expression for indicated circadian genes (numerator) over the housekeeping genes *18s* and *Actin (denominator)*. All data are presented as mean ± SEM. P<0.05 was considered statistically significant by Mann-Whitney U test for (A-D) and by 2-way ANOVA with Bonferroni correction for (E). * indicates statistically significant effect of Zeitgeber time points between control and MLP offspring groups.

To examine if circadian rhythm abnormalities are at the origin of the altered rest/sleep pattern described above, we carried out circadian behavior testing. At the end of the two-week light:dark (L:D) acclimation phase, (Fig 3A; top part), wheel running activity was not significantly different between control and MLP offspring (P = 0.56) (Fig 3B). As shown in the actogram, exposure to constant darkness (D: D) elicited negligible phase advancement with intact rhythmicity in both groups (Fig 3A, lower part). Both groups exhibited similar wheel running activity in D:D conditions (P = 0.39) (Fig 3C). We analyzed the periodicity (Tau values) between MLP and control offspring using the F test for variances. We did not find any variability in the distribution of the Tau values between MLP and control offspring (P = 0.71) (Fig 3D).

Multiple studies have linked food intake with circadian transcriptome changes [41, 42]. Thus, we examined if exposure to the low protein diet could still result in subtle differences in expression of the circadian genes in hypothalamus as a potential contributor to the observed increased body fat and decreased EE We chose the hypothalamus for analysis of circadian gene expression in MLP-exposed and control offspring, because it is the location of the central circadian clock. We analyzed circadian gene expression using two different housekeeping genes, *18s rRNA* and *Actin* owing to their stability and non-oscillatory expression. As expected, we found a statistically significant Zeitgeber time effect in *Per1* expression relative to *18s*, *Clock* and *Per2* expression relative to *Actin* between the two groups (Fig 3E). There was no significant impact of diet on circadian gene expression. Taken together, the data from wheel running assay and the gene expression analysis indicate that there is no effect of low protein diet on circadian gene expression in the hypothalamus.

### Chronic MLP diet-exposed male offspring show anxiety-like behavior and a sociability deficit but no depression-like or repetitive behaviors

Studies have shown that dietary modifications during gestation can influence neurodevelopment in male [14, 15] and female [14] offspring, manifesting as behavioral changes. Therefore, we carried out a battery of neurobehavioral assays for anxiety, depression, repetitive behaviors, sociability, and learning and memory. In particular, because we did not observe circadian rhythm disturbances, we wanted to examine if MLP-exposed offspring had a depression-like behavioral state as an alternate explanation for the reduced activity during the active phase.

**Fig 4:**
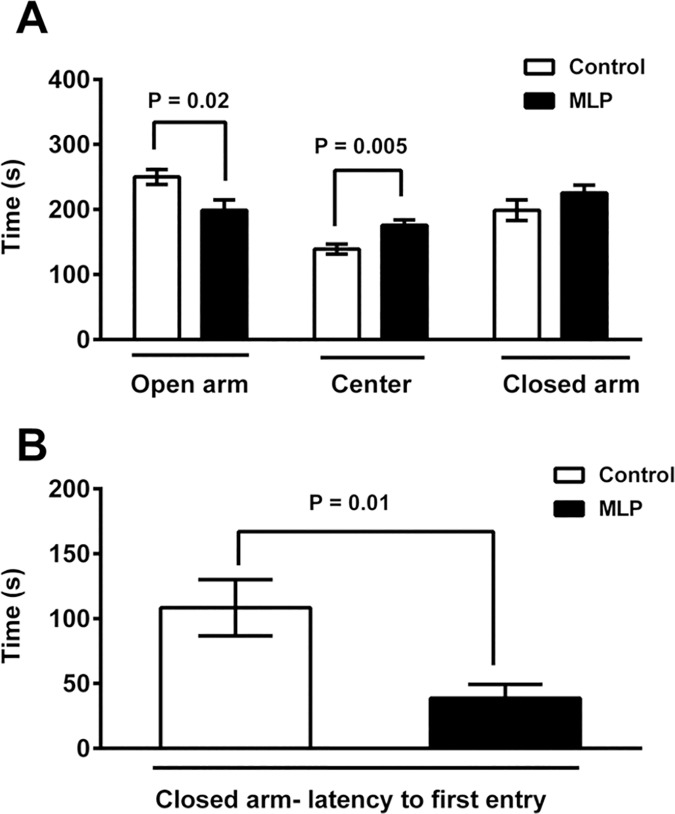
MLP male offspring mice display anxiety-like but not depression-like behavior. Elevated plus maze test (MLP and Control, n = 9 each); (A) Time spent in open arm, center and closed arm, (B) Latency to enter closed arm. (C) Tail suspension test (MLP, n = 31 and Control, n = 13) and (D) Forced swim test (MLP and Control, n = 20 each). Data is presented as mean ± SEM. P<0.05 was considered statistically significant by student t-test.

There was no significant difference between MLP and control offspring in any of the tested parameters in the open field assay (S1 Fig). In the elevated plus maze (EPM) test for anxiety-like behavior [43], the MLP-exposed offspring spent significantly less time in the open arm (P = 0.02) and longer time in the center (P = 0.005) (Fig 4A). The MLP offspring also entered the closed arm (P = 0.01) faster than control mice (Fig 4B), but the total time spent in the closed arm was not different between the two groups (Fig 4A). The reduced time spent in the open arm and shorter latency to first closed arm entry indicates that MLP offspring may be more anxious, but the anxiety phenotype was incompletely manifest because they did not spend more total time in the closed arm.

To investigate the potential anxiety-like phenotype observed in the EPM more completely, we next performed the light/dark exploration assay, but saw no significant difference between the two groups in latency to enter the dark compartment (P = 0.79) (S2 Fig), time spent in the light compartment (P = 0.19) (S2 Fig), and number of transitions between light and dark compartments (P = 0.28) (S2 Fig). Thus, although partial data from the EPM test suggest that the MLP offspring are more anxious, we did not observe increased anxiety in the open field assay and the light/dark exploration assay.

Because anxiety-like traits were modest and MLP offspring also displayed increased resting/sleeping during the dark phase (Fig 2), we examined if they displayed depression-like behavior by performing the tail suspension test (TST) and the forced swim test (FST). There was no difference in immobility time in the TST between MLP offspring (118±11 seconds) and control offspring (148±11 seconds) (Fig 4C, P = 0.12). In the FST, the MLP offspring did not display increased immobility time (20±6 seconds) compared to control offspring (30±6 seconds) (Fig 4D, P = 0.5). These results indicate that the MLP offspring did not display more depression-like behavior compared to controls.

**Fig 5:**
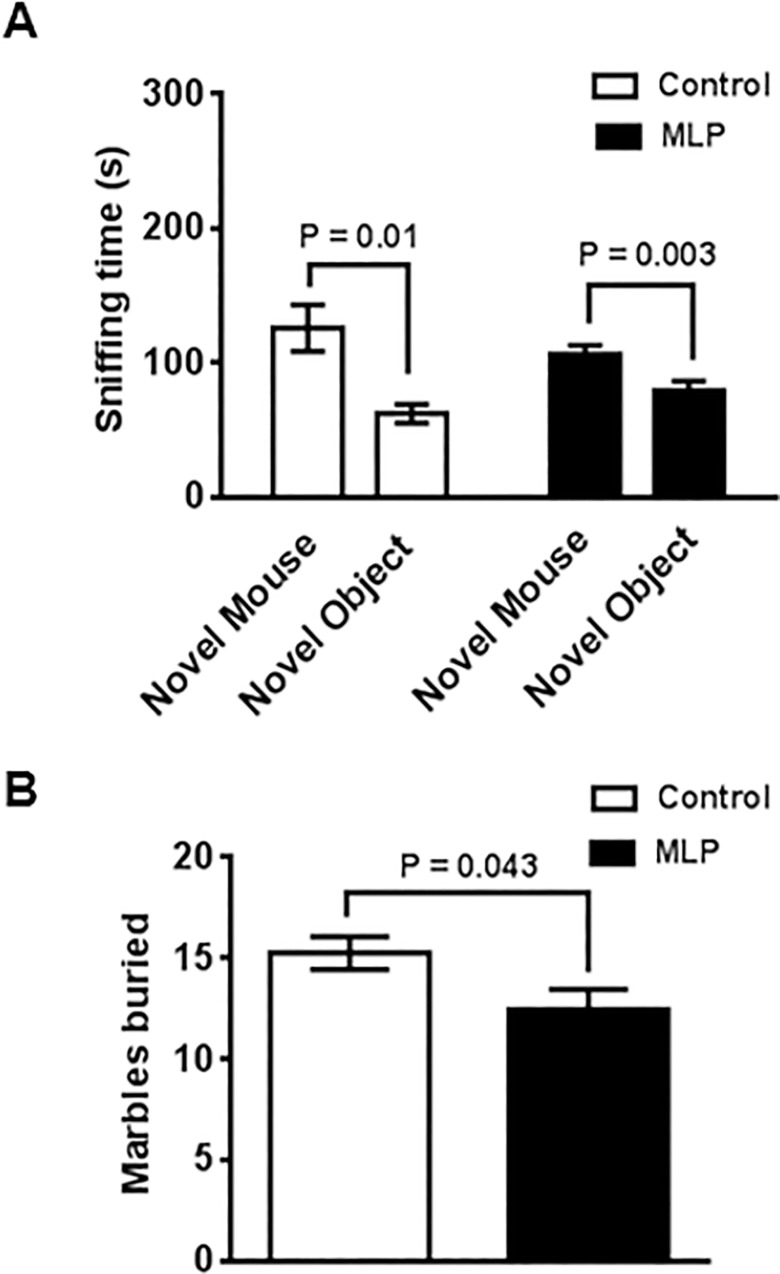
MLP male offspring mice have no sociability phenotype and display decreased repetitive behavior. (A) Three-chamber test (MLP and Control, n = 9 each), (B) Marble burying (MLP and Control, n = 9 each). Data is presented as mean ± SEM. P<0.05 was considered statistically significant by student t-test for (A) and (B).

**Fig 6:**
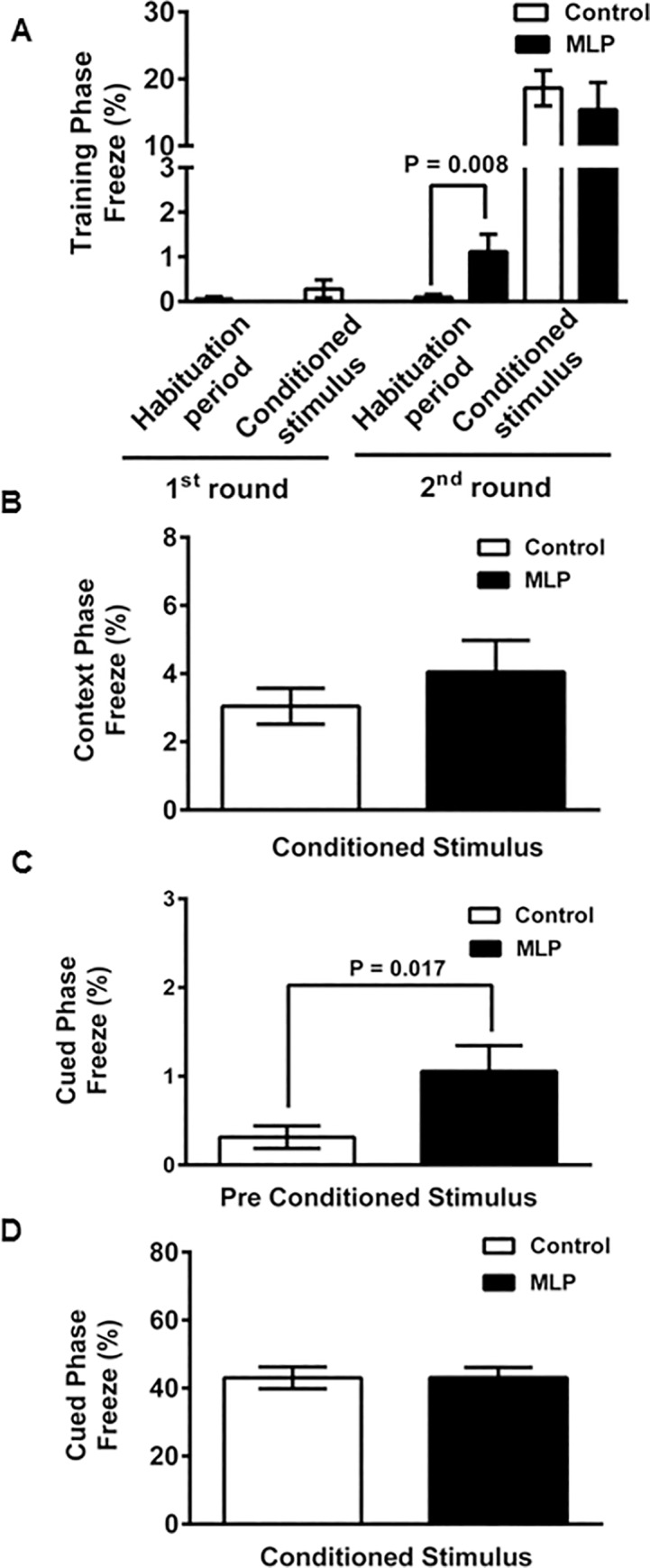
MLP male offspring mice show anxiety to an unfamiliar environment but no associative learning and memory deficit. Fear conditioning test in MLP and Control male offspring mice. (A) Training phase (MLP and Control, n = 20 each), (B) Context phase, (C) pre cue phase, and (D) cue phase. MLP, n = 18; Control, n = 19 for (B-D). Data is presented as mean ± SEM. P<0.05 was considered statistically significant by Mann Whitney test.

We next evaluated sociability, repetitive behavior, and associative learning and memory of the MLP and control offspring by the three chamber (3CH), marble burying and fear conditioning test, respectively. As shown in Fig 5A, both MLP and control offspring spent significantly more time sniffing the cup with a novel mouse than the empty cup, as expected (MLP, P = 0.003; Control, P = 0.01) in the 3CH test, indicating that MLP offspring do not have a sociability deficit. In the marble-burying test for repetitive and pervasive behaviors [34], MLP offspring buried significantly fewer marbles (P = 0.04) than control offspring, indicating abnormally decreased repetitive behavior (Fig 5B). In the fear-conditioning (FC) assay, the MLP offspring froze more (P = 0.008) during the second habituation period on the training day, likely consistent with the observed anxiety in the EPM. However, the second training conditioned stimulus (CS) (sound cue followed by footshock) elicited the same response in both groups (P = 0.51) (Fig 6A). During the contextual memory testing on day 2, the MLP and control offspring displayed similar freezing when placed back in the same cage (Context CS) (P = 0.30) (Fig 6B). During cued fear conditioning (pre CS), the MLP offspring froze more when placed in the new environment (P = 0.017) (Fig 6C) but there was no difference in freezing to the auditory cue (cue CS) (P = 0.99) (Fig 6C). These data were indicative of hyperanxiety when presented with an unfamiliar environment but no deficit in contextual or cued learning and memory.

Taken together, the above data suggests that exposure to chronic MLP diet may affect offspring behavior, manifested as some increase in anxiety but no deficit in learning and memory. We also did not find objective evidence of depression-like behaviors or deficits in sociability.

## Discussion

The primary objective of this study was to comprehensively characterize the metabolic consequences, neurobehavioral and circadian rhythm effects on male offspring of C57/Bl6J dams chronically exposed to a protein-restricted diet from four weeks before gestation and continued throughout lactation. Previously, we have shown in this model that male MLP offspring have reduced body weight gain compared to controls [27]. However, in contrast to some other models with exposures to MLP only from the beginning until the end of pregnancy [44–46], the offspring did not catch-up in weight but remained small with a normal growth rate. They also did not have impaired glucose tolerance or any overt pathology in response to developmental exposure to chronic MLP diet [27]. In the present study, we extended these observations by examining whole body fat and lean content. While we did not observe differences in weight at 12 weeks of age, paradoxically, we found that a higher proportion of the measured body weight of these offspring was derived from fat indicating that the MLP offspring were in a positive energy balance (Fig 1). This is also consistent with our previous observations that muscle mass was reduced in these mice [27]. We did not examine maternal weight in our study. However, a recent report presents data that dams fed with low protein diet had reduced body weight mid and late gestation [47].

To evaluate the cause of body composition difference, we measured the major components of energy balance. There was no difference in food intake between MLP and control offspring, but there was a decrease in total daily energy expenditure (EE), that occurred during the dark phase (Table 1) which is typically the most active time for mice. We also noted that the respiratory exchange ratio (RER) during the night was lower in MLP offspring (Table 1). This could reflect the tendency for MLP mice to eat less of the high carbohydrate chow than the control mice during the dark phase. Regardless, a difference of <3% is of questionable biological significance. The decrease in EE, together with the high fat accumulation, strongly suggests that the chronic nutrient restriction during development has programmed an overall reduction in total EE. Without an equivalent decrease in energy intake, this would result in a positive energy balance. Estimates of energy balance over a full 3 day measurement period were not significantly different (data not shown), but this was not surprising given the inherent error associated with the measurement and that differences in fat deposition likely result from cumulative differences in energy balance over the lifetime of the animal.

To understand the differences in EE between groups, we considered the various components of EE. Activity and resting EE make the largest contribution to total EE. We did not measure resting EE in this study and, therefore, cannot be certain whether it may have contributed to the difference in total EE between groups. However, under the experimental conditions of this study, differences in resting EE would exhibit minimal circadian variation and, thus, are unlikely to account for the observed decrease in EE of the MLP group which mostly occurred in the dark phase. The evaluation of activity patterns and sleep patterns, however, were more insightful. MLP offspring displayed more hypoactive behaviors in the CLAMS only during the dark/active phase, manifested as a reduction in rearing, and an increase in time spent inactive, i.e., resting/sleeping, (Fig 2). Previous studies in rats have demonstrated that the adult offspring of dams fed an MLP diet during gestation have altered sleep architecture: during the natural sleep cycle these differences are not reflected in total time spent awake [48], but they spend more time awake during a part of the dark cycle [49]. However, an effect of the MLP diet during gestation on measures of 24 hr spontaneous activity have identified no corroborating evidence of this altered sleep/wake cycle [50]. Although these reported observations differ from ours, the studies also differ in several important respects: for example our data are from offspring mice whose dams were exposed to MLP from prior to gestation and during lactation; our method of quantifying wakefulness/sleep also differed. Whereas other studies used telemetric EEG and EMG monitoring, the primary difference we observed was in the z (vertical)-dimension which may not be registered when using telemetric devices. However, because the behavioral differences we observed may have contributed to the differences in total EE, further studies to identify the significance of the decreased wakefulness in the MLP offspring are warranted.

Because the largest discrepancy in total EE between groups occurred at a time associated with lower activity, we explored which behavioral abnormalities might underlie these observations. Specifically, we set out to examine if the MLP offspring had a disrupted circadian rhythm or altered neurobehavior, such as a depressive-like state or anxiety. Therefore, we performed comprehensive set of neurobehavioral assays for depression like features, anxiety, sociability, and learning and memory.

We did not find circadian rhythm abnormalities as measured by wheel-running activities in the L:D, D:D test (Fig 3A, Fig 3B, Fig 3C and Fig 3D). We only found a time-effect difference in expression of *Per* 1, *Clock* and *Per 2* in the hypothalamus of MLP offspring compared to Control offspring (Fig 3E). The absence of circadian gene expression changes is consistent with the observation that MLP offspring show no circadian rhythm abnormality. However, the hypothalamus does not complete maturation until the second week of postnatal life in rodents, and thus the timing and duration of exposure of the offspring to a nutritional insult can have profoundly different outcomes [51, 52]. We also note that the circadian gene expression analysis was done on whole hypothalamus. Considering that *Clock* levels can vary in various hypothalamic regions [53], future experiments could focus on the suprachiasmatic nucleus (SCN) by using RNA extracted from tissue punches for gene expression analysis by qRT-PCR and immunocytochemistry-based protein expression studies focused on regions of the SCN. It is intriguing that the increased night-time resting/sleeping state of MLP-exposed offspring compared to control mice (Fig 2A) was not reflected in the wheel running assay during housing in constant darkness (Fig 3C). We note that different types of behavior are tested in CLAMS (including unconscious passive and active behavior), compared to circadian wheel running assay, which only measures active behavior. Although we have no explanation for this observation, it shows that the effects of early exposure to a suboptimal maternal nutritional state are complex and can vary with alterations in the adult environment. A large number of behavioral and physiological pathways exhibit a circadian rhythm. The rhythms of these pathways are regulated by molecular clocks in various cell types, all of which are regulated by the central circadian clock in the SCN. One study demonstrated that MLP significantly altered the circadian expression of genes involved in the regulation of feeding and energy metabolism [21]. In addition, supplementation with the essential amino acid tryptophan has been shown to improve circadian function of cells isolated from MLP offspring [54]. Furthermore, offspring exposed to a different maternal dietary manipulation (maternal high fat diet) show changes in core circadian gene expression [55]. Despite the suggestion of a potential impact of MLP on offspring circadian behavior, a comprehensive analysis of circadian behavior, as well as the peripheral and central molecular clock machinery has been relatively unexplored. To our knowledge, this study is the first to comprehensively evaluate the effects of chronic MLP on offspring circadian behavior and molecular clock machinery.

We found a modest effect on offspring anxiety (Fig 4A and 4B) and reduced repetitive behaviors (Fig 5B) that was assay-dependent. This is not surprising as it is known that behavioral responses, such as anxiety, may be high in one assay and low in another [56]. Studies have also shown that short-term dietary tryptophan depletion affects obsessive compulsive behavior in humans [57, 58].

Although our findings were more subtle, a stronger anxiety and/or depressive phenotype in MLP offspring in the CF1 strain of mice [14] and in the more commonly used rat model has been shown [15]. It is known that when comparing behavioral outcomes from different studies, the species and genetic background, as well as the content and exposure timing and length of the MLP diet can have dramatic effects on the severity of the behavioral phenotype of the offspring [2, 11, 13, 15]. Indeed, a few reports suggest that exposure to MLP during the later stages of gestation and lactation results in an overt behavioral effect, while MLP exposure during the entire gestation period and lactation had variable effects similar to our results [15]. In the current study, virgin female C57BL/6J mice consumed a low-protein diet beginning 4-weeks prior to mating until the end of lactation which is a more relevant model of a chronically malnourished population rather than only gestational MLP. We speculate that these dams may have had more time to adapt metabolically to their suboptimal diet and, therefore, offspring may have been partially protected from acute maternal low-protein malnutrition.

Housing conditions can also drastically impact behavior. In this model, offspring from both groups were continually, single-housed beginning at weaning (day 21 of age) to have a more controlled environment for individual food intake standardization, but mice are naturally social animals, and previous studies have demonstrated that chronic exposure to single housing, even with environmental enrichment, can by itself alter behavior [59–61]. Single-housed mice have been demonstrated to show reduced freezing in a novel context [60, 61]. Our observation is in agreement with this, but we saw a significantly heightened response in MLP offspring to a novel context compared to control offspring prior to administration of cues which equalized again upon introducing the cues. Although the housing conditions may have affected the behavioral responses, no differences in these responses were observed between the MLP and control groups, supporting that there was no interaction between the effects of environment and the MLP exposure. While we only examined male offspring from dams exposed to low protein diet, female offspring can also be developmentally affected [62] and should be part of future experiments. Maternal diet can influence offspring development [9, 62] and in this context, it will be interesting to examine hormonal profiles in these offspring.

## Conclusion

In this mouse model of exposure to chronic maternal protein malnutrition, we have demonstrated that offspring are smaller, but have higher body fat content. This difference in body composition was in conjunction with lower levels of EE and nocturnal hypoactivity during the normally active time for mice. This difference was not explained by circadian rhythm alterations, but the mice exhibited mild anxiety-related behavioral differences along with nocturnal hypoactivity. Future studies will focus on epigenetic, neuroanatomical and neurophysiological correlates of these effects, which can uncover potential future therapeutic targets that can modify this complex phenotype.

## Supporting Information

S1 Fig Open field activity in MLP and Control male offspring mice. MLP and Control offspring (18–20 weeks age, n = 9 each) examined by open field test. The test examined (A) total distance traveled; (B) center distance traveled; (C) total time moving; (D) speed of mobility; (E) stereotypy; (F) vertical activity; (G) total revolutions. Data is presented as mean ± SEM with P<0.05 considered statistically significant by student t-test.
(DOCX)

S2 Fig Light/dark exploration test in MLP and Control male offspring mice. MLP and Control offspring (18–20 weeks age, n = 20 each) tested in light/dark exploration assay. Data presented are (A) latency to enter dark chamber, (B) time spent in light chamber, and, (C) total transitions between light and dark chambers. Data shown is mean ± SEM with P<0.05 considered statistically significant by student t-test.
(DOCX)

S1 Table Mouse circadian gene q-RTPCR primer sequences. (DOCX)
