# Correction: Chronic Maternal Low-Protein Diet in Mice Affects Anxiety, Night-Time Energy Expenditure and Sleep Patterns, but Not Circadian Rhythm in Male Offspring

**DOI:** 10.1371/journal.pone.0201079

**Published:** 2018-07-17

**Authors:** Randy F. Crossland, Alfred Balasa, Rajesh Ramakrishnan, Sangeetha K. Mahadevan, Marta L. Fiorotto, Ignatia B. Van den Veyver

In Fig 4, panels C and D are missing. Please see the corrected Fig 4 here.

**Fig 4:**
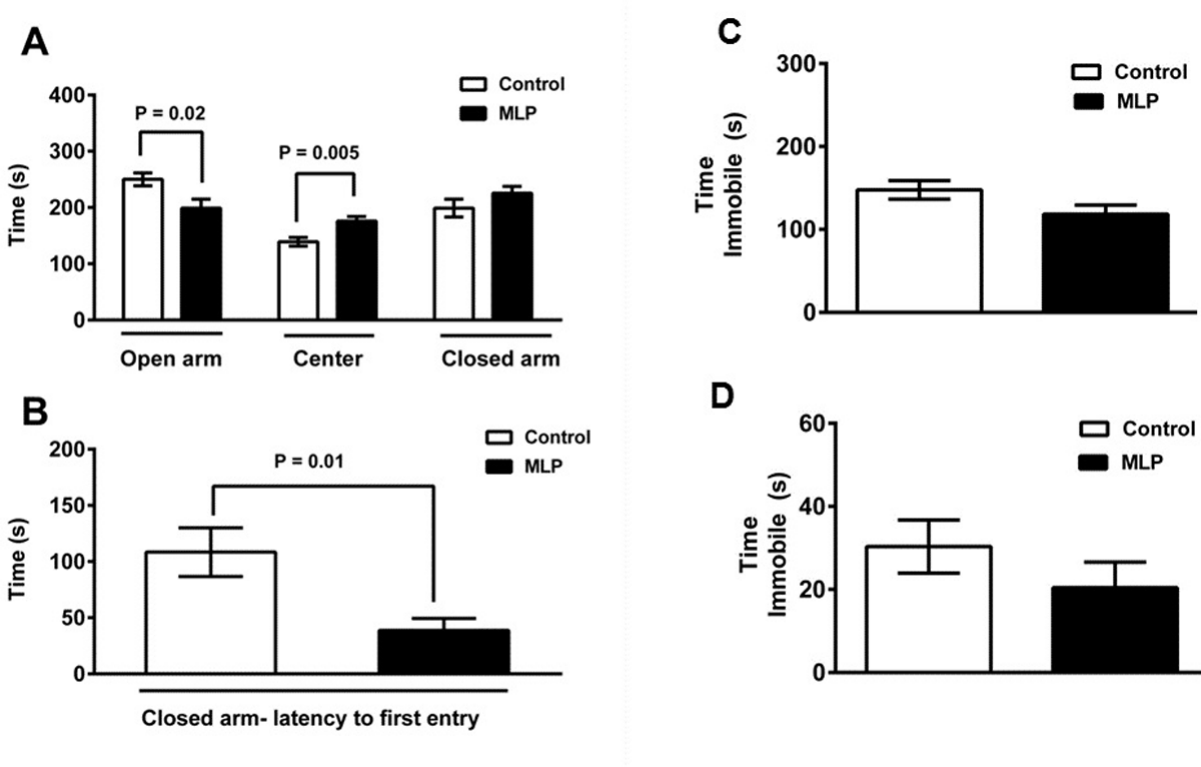
MLP male offspring mice display anxiety-like but not depression-like behavior. Elevated plus maze test (MLP and Control, n = 9 each); (A) Time spent in open arm, center and closed arm, (B) Latency to enter closed arm. (C) Tail suspension test (MLP, n = 31 and Control, n = 13) and (D) Forced swim test (MLP and Control, n = 20 each). Data is presented as mean ± SEM. P<0.05 was considered statistically significant by student t-test.
