# Supplementary material for: Chronic Maternal Low-Protein Diet in Mice Affects Anxiety, Night-Time Energy Expenditure and Sleep Patterns, but Not Circadian Rhythm in Male Offspring
Source: PLoS One. 2017 Jan 18;12(1):e0170127. doi: 10.1371/journal.pone.0170127 (PMC5242516; doi:10.1371/journal.pone.0170127)
Supplement: S1 Fig — MLP and Control offspring (18–20 weeks age, n = 9 each) examined by open field test. The test examined (A) total distance traveled; (B) center distance traveled; (C) total time moving; (D) speed of mobility; (E) stereotypy; (F) vertical activity; (G) total revolutions. Data is presented as mean ± SEM with P<0.05 considered statistically significant by student t-test. (DOCX) [file pone.0170127.s001.docx]

**S1 Fig.
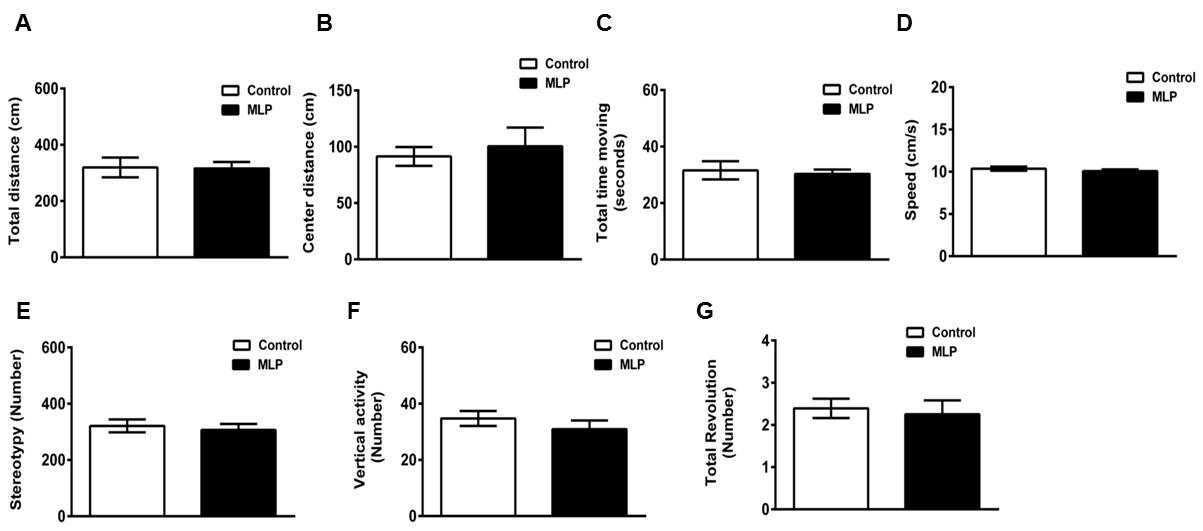
**

**S1 Fig. Open field activity in MLP and Control male offspring mice.**  MLP and Control offspring (18-20 weeks age, n=9 each) examined by open field test. The test examined (A) total distance traveled; (B) center distance traveled; (C) total time moving; (D) speed of mobility; (E) stereotypy; (F) vertical activity; (G) total revolutions. Data is presented as mean ± SEM with P<0.05 considered statistically significant by student t-test.
