# Supplementary material for: Chronic Maternal Low-Protein Diet in Mice Affects Anxiety, Night-Time Energy Expenditure and Sleep Patterns, but Not Circadian Rhythm in Male Offspring
Source: PLoS One. 2017 Jan 18;12(1):e0170127. doi: 10.1371/journal.pone.0170127 (PMC5242516; doi:10.1371/journal.pone.0170127)
Supplement: S2 Fig — MLP and Control offspring (18–20 weeks age, n = 20 each) tested in light/dark exploration assay. Data presented are (A) latency to enter dark chamber, (B) time spent in light chamber, and, (C) total transitions between light and dark chambers. Data shown is mean ± SEM with P<0.05 considered statistically significant by student t-test. (DOCX) [file pone.0170127.s002.docx]

**
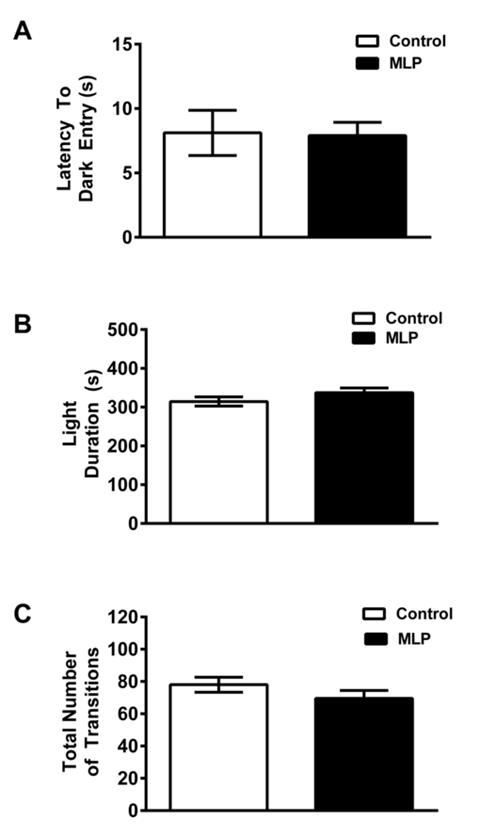
 S2 Fig.**

**S2 Fig. Light/dark exploration test in MLP and Control male offspring mice.**  MLP and Control offspring (18-20 weeks age, n= 20 each) tested in light/dark exploration assay. Data presented are (A) latency to enter dark chamber, (B) time spent in light chamber, and, (C) total transitions between light and dark chambers. Data shown is mean ± SEM with P<0.05 considered statistically significant by student t-test.
