# Supplementary material for: Chronic Maternal Low-Protein Diet in Mice Affects Anxiety, Night-Time Energy Expenditure and Sleep Patterns, but Not Circadian Rhythm in Male Offspring
Source: PLoS One. 2017 Jan 18;12(1):e0170127. doi: 10.1371/journal.pone.0170127 (PMC5242516; doi:10.1371/journal.pone.0170127)
Supplement: S1 Table — (DOCX) [file pone.0170127.s003.docx]

**S1 Table. Mouse circadian gene q-RTPCR primer sequences.**
